# Sepsis requiring intensive care following intramuscular injections: two case reports

**DOI:** 10.4076/1757-1626-2-7365

**Published:** 2009-08-18

**Authors:** Dimitrios Velissaris, Charis Matzaroglou, Christina Kalogeropoulou, Vassilios Karamouzos, Kriton Filos, Menelaos Karanikolas

**Affiliations:** 1 Department of Anaesthesiology and Critical Care Medicine, Patras University HospitalRion 26500Greece; 2 Department of Orthopaedic Surgery, Patras University HospitalRion 26500Greece; 3 Department of Radiology, Patras University HospitalRion 26500Greece

## Abstract

**Introduction:**

Intramuscular injections can rarely result in serious infectious complications such as abscesses which may progress to bacteraemia and generalized sepsis. These complications are rare, but can be life threatening, as they can lead to multi-organ failure associated with high morbidity and mortality.

**Case presentation:**

In this report we present two patients who developed life-threatening infections after intramuscular injections. They were admitted to the hospital, had prompt surgical drainage, required ICU admission for severe sepsis, were treated with an early goal-directed therapy protocol and had a good outcome.

**Conclusion:**

Sepsis is a rare, potentially life-threatening complication after intramuscular injections. Timely surgical drainage followed by appropriate ICU care and early goal directed therapy is crucial and may contribute to a good outcome in these rare cases.

## Introduction

Intramuscular injections (IM) can lead to local infectious complications, such as abscesses [1,2] skin necrosis [3] or intra-articular infections, and can rarely progress to generalized sepsis and multi-organ failure [4]. Such serious infections are more likely to occur in immuno-compromised patients but have also been described in immune-competent persons. They are usually caused by *Staphylococcus aureus* and require timely medical and surgical treatment. Life-threatening generalized sepsis is rare but has been reported [4,5], and aggressive care in an ICU is required for optimal results in these cases. We describe two patients with multiple abscesses and generalized sepsis caused by *Staphylococcus aureus* after intramuscular injections. Both patients were treated with early surgical drainage, were supported in the ICU with a goal-directed protocol [6] and had a good outcome.

## Case presentation

### Case report 1

**Figure 1.**
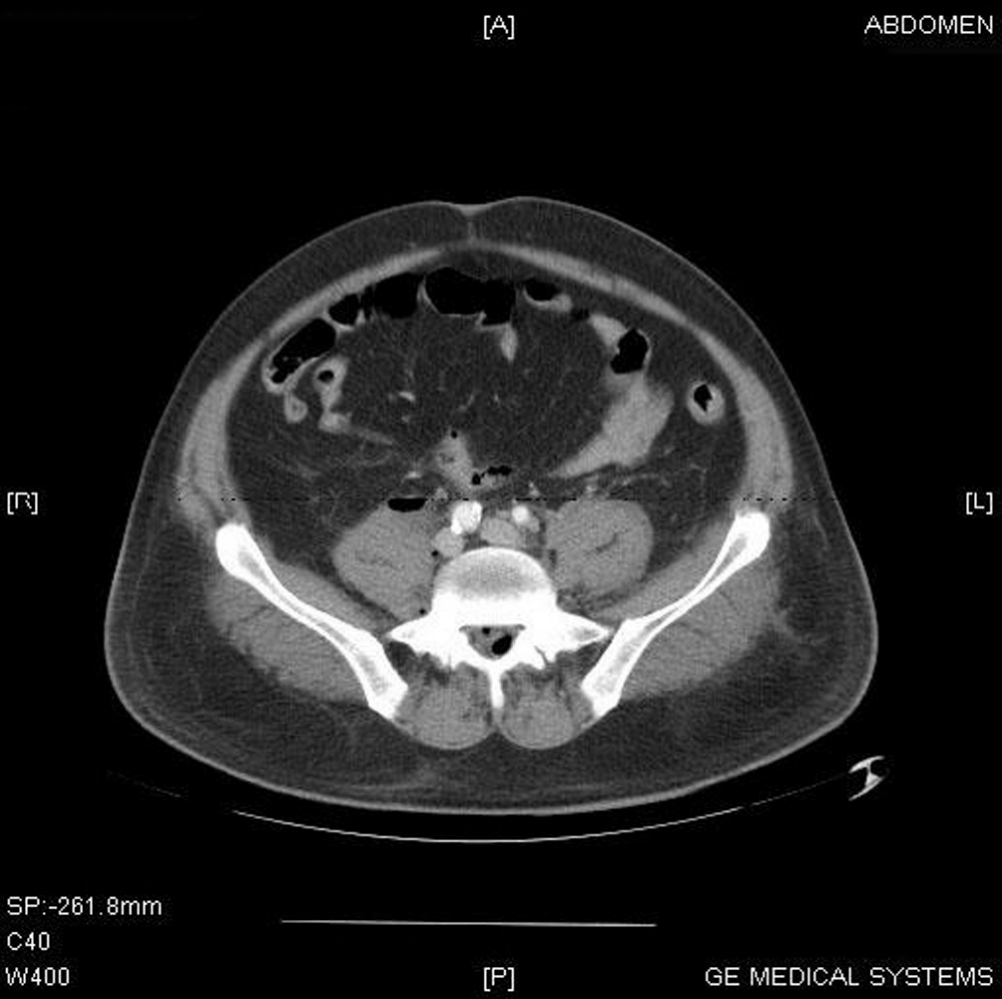
CT of the abdomen demonstrating extensive air and fluid involving the fascias along the psoas muscles, and air along the epidural space of the spinal canal in the lumbar region.

**Figure 2.**
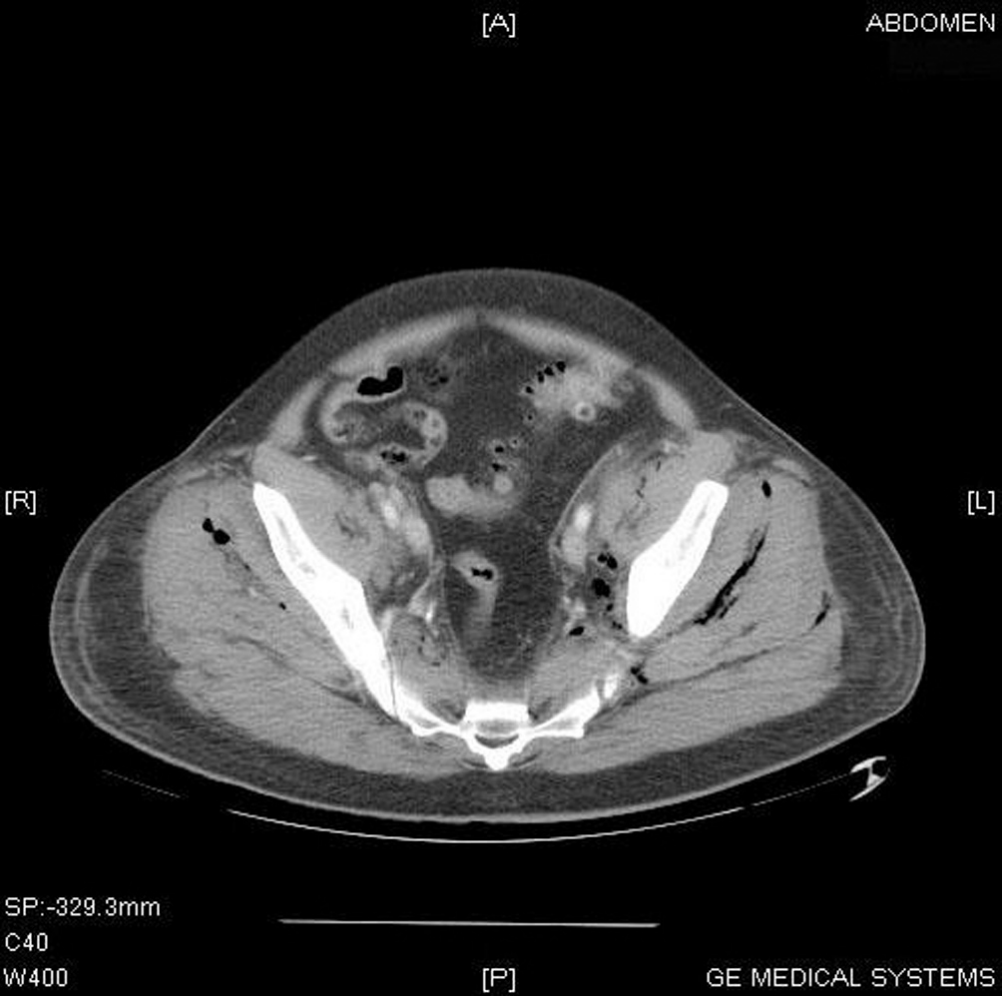
CT of the pelvis demonstrating extensive air and fluid extending through the pelvic fascias to the pelvic muscles.

**Figure 3.**
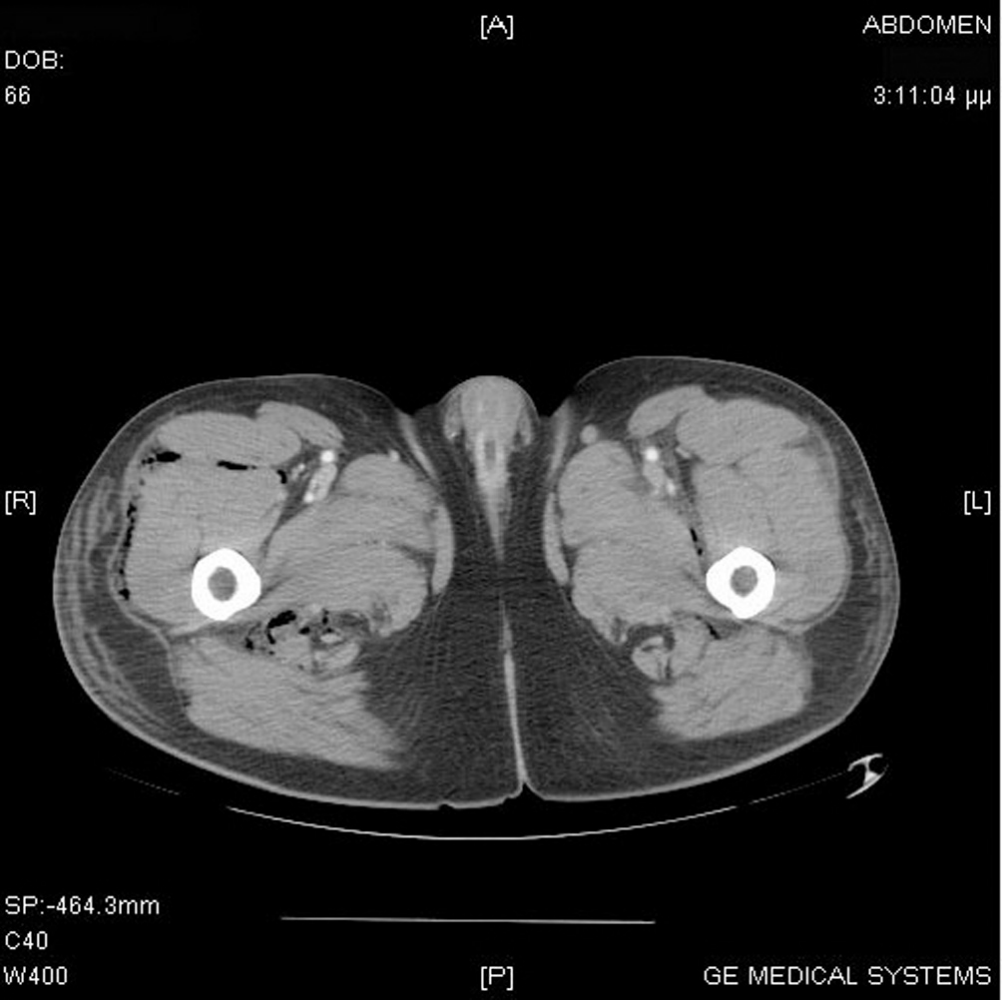
CT of the thighs revealing skin thickening, increased subcutaneous fat attenuation and subcutaneous and inter-muscular fat stranding in the right thigh.

A 54-year-old Caucasian male patient with insulin-dependent diabetes and remote history of alcohol abuse was admitted through the Emergency Room with high fever. Physical examination revealed a small tender, erythematous, fluctuant mass on the antero-lateral aspect of the right thigh. He had received just one IM aminoglycoside injection for treatment of an upper respiratory infection, approximately 48 hours before coming to the Hospital. Diagnostic workup [7,8] included CT of the abdomen and thighs with and without contrast, which revealed air and fluid collections in the peritoneum and right thigh (Figures 1, 2, 3). The patient had retroperitoneal abscess drainage via bilateral J-shaped lower abdominal incision and right thigh abscess drainage via a Moore-type southern incision in the operating room. After the wound was closed, drains were left for continuous retroperitoneal space irrigation. Postoperatively, the patient was transferred to the ICU and was supported with a goal-directed therapy protocol [6]. Broad-spectrum antibiotic therapy consisted of Meropenem (500 mg every 8 h), Vancomycin (500 mg twice daily), Gentamicin (120 mg once daily) and Fluconazole (400 mg once daily). Due to hemodynamic instability, dopamine 10 mcg/Kg/min was administered for 7 days. He also received Hydrocortisone 300 mg/day IV for 6 days, and continuous IV insulin infusion to keep blood glucose <140 mg/dl. Weaning from mechanical ventilation was successful on ICU day 9, after bilateral infiltrations on CXR improved. He developed renal dysfunction (maximum serum creatinine was 2.4) but urine output remained adequate (> 0.5 ml/kg/h) and dialysis was not needed. Multi-resistant *Staphylococcus aureus* was isolated from blood cultures. The patient remained in the ICU for 12 days, and was then transferred in stable condition to the Surgery Ward, his general condition gradually improved and he was discharged home in good condition.

### Case report 2

**Figure 4.**
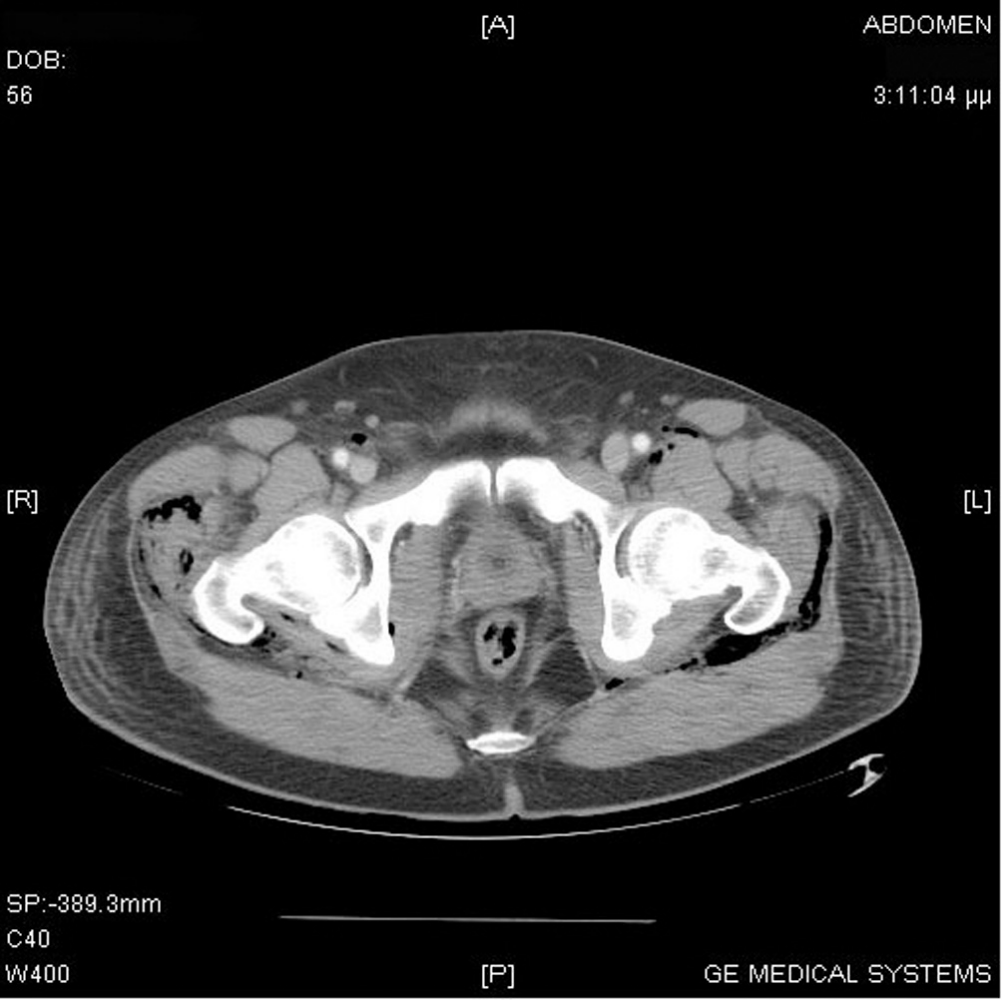
CT of the pelvis and lower extremities demonstrating extensive air and fluid through the upper part of the thighs. Air and fluid was also depicted along the fascias of the muscles on both buttocks.

A 73 year old Caucasian female patient with history of hypertension, congenital hip dislocation, and degenerative arthritis, was admitted to the Internal Medicine Ward due to anemia (Hb:5.7 gm/dL), persistent low-grade fever (37.3°C) and lower extremity arthralgia. The main finding on physical examination was a painful, warm, fluctuant mass with marked skin erythema and necrosis in the gluteal area. She had received many Non Steroidal Anti-inflammatory Drug (NSAID) IM injections for arthritis pain over the last several months. Diagnostic workup included a CT-scan of the abdomen, which revealed fluid collection along the soft tissues on both thighs, extending into the abdominal cavity. Large intra-peritoneal fluid collections were recognized on both sides of the bladder, and smaller fluid collections were present along both femoral heads within the gluteal muscles bilaterally (Figure 4). The patient had intra-peritoneal abscess drainage through a J-shaped lower abdominal incision, followed by drainage of the femoral fluid collections in the operating room. Two soft drainage tubes were inserted through the abdominal wall, drains were left in place on both thighs, and the surgical wound was closed. Postoperatively, the patient was admitted to the ICU due to developing multi-organ failure, (tachycardia, hypotension, oliguria and deterioration of pulmonary function). In the ICU she required mechanical ventilation and was supported with a goal-directed therapy protocol [6] as described in case one. Broad-spectrum antibiotic therapy included Vancomycin 500 mg twice daily, Meropenem 500 mg three times daily and Fluconazole 400 mg once daily. *Staphylococcus aureus* was isolated from blood cultures. Adequate urine output was maintained with continuous IV furosemide infusion at 2 mg/h for 7 days. Total ICU length of stay was 15 days, and she then transferred to the orthopaedic surgery ward in stable condition, gradually improved and was discharged home.

Demographics and medical history and ICU data are presented in Table 1.

**Table 1.** Demographics, medical history and ICU data

Case	Age / Sex	Medical History	Drug injected IM	Admission SOFA score	Mechanical Ventilation	Renal Failure/ Dialysis	Inotropic Support	ICU stay	Outcome
1	54 / Male	Alcohol abuse, diabetes	Aminoglycoside	10	9 days	Yes/No	Dopamine 7 days	12 days	Good
2	73 / Female	Hypertension, Arthritis	NSAID	10	10 days	Yes/No	Dopamine 5 days	15 days	Good

## Discussion

An intramuscular (IM) injection is a minor procedure whereby a drug is deposited into a muscle via a sterile needle. Most IM injections should be performed into the deltoid muscle in the arm, or into the gluteus maximus muscle in the buttocks. Recent studies [2,3] have highlighted the importance of correct IM drug administration, in order to minimize the risk of potentially serious complications. Appropriate clinical practice needs to reflect considerations about appropriate needle length and gauge, to ensure that patients get the benefit of drug administration without adverse effects. Muscle tissue is usually spared the harmful effects of substances injected into it, probably because of its abundant blood supply. However, deep IM injections can cause abscesses and granulomas, whereas more superficial IM injections may result in increased incidence of local reactions, such as irritation, inflammation and necrosis [1,2,9].

In this report we present two patients who developed sepsis and multiple organ failure after IM injections. Injection safety is a complex problem, and unsafe practices can place patients at increased risk of infection [3,6]. However, even when properly administered, IM injections can result in severe tissue trauma, by creating a local entry point for bacteria. Although aminoglycosides are often administered as IM injections, we could not find any published reports of necrotizing fasciitis or other serious local infection after aminoglycoside injections. In contrast, several reports of serious or fatal complications from NSAID injections have been published [3,10-12].

Sepsis and multi-organ failure are the leading causes of death in critically ill patients [13]. Much of modern critical care practice is based, in principle, on restoring aberrant respiratory, cardiovascular and other functions to physiologic levels in an attempt to maintain or restore adequate organ perfusion. Goal-directed therapy has been used for severe sepsis and multiple organ dysfunction in the Intensive Care Unit [6], and consists of maintaining CVP > 8-12 mmHg, MAP > 65 mmHg, urine output > 0.5 ml/kg/h, SvO2 > 70%, SaO2 > 93% and hematocrit > 30%. Both patients required mechanical ventilation and received broad-spectrum antibiotics, IV fluids and pharmaceutical agents (dopamine) to achieve and maintain the aforementioned physiologic goals.

These two cases highlight the potential for severe tissue necrosis following IM injections. The pathogenesis explaining skin necrosis is uncertain, but damage to an end-artery is a plausible hypothesis. Other causes to consider, especially when deep tissue necrosis is also present, are the cytotoxic effect of the drug or additives in the injectate. Both our patients had skin necrosis that progressed to bacteremia and sepsis from multi-resistant *Staphylococcus aureus*, a finding consistent with previously published cases [1,2].

Serious complications, including limb amputation and life-threatening sepsis related to IM injections have been reported in the past [1-5]. However, we believe this report is an important addition to previously reported cases of sepsis and multi-organ failure following IM injections, because both patients became severely ill (SOFA [14] score 10 on ICU admission) and were treated with early surgical intervention [5], followed by prolonged ICU care with a goal-directed therapy protocol [6]. We believe that timely radical surgical intervention followed by goal-directed ICU care probably contributed to the good outcome in both cases.

## Conclusion

Minor medical interventions, such as intramuscular injections, can rarely result in life-threatening infections. If a serious infection occurs, isolation of the offending pathogen is helpful in ensuring that appropriate antibiotic therapy is given. Timely radical surgical intervention with abscess drainage is crucial and may help improve outcome. Goal directed therapy in the ICU may contribute to a good outcome in patients who develop sepsis and multi-organ failure.

## Abbreviations

CT: computed tomography
ICU: intensive care unit
IM: intramuscular injections
NSAID: non-steroidal anti-inflammatory drug
